# Supplementary material for: Comprehensive Analysis of the Green-to-Blue Photoconversion of Full-Length Cyanobacteriochrome Tlr0924
Source: Biophys J. 2014 Nov 4;107(9):2195–203. doi: 10.1016/j.bpj.2014.09.020 (PMC4223177; doi:10.1016/j.bpj.2014.09.020)
Supplement: Document S1. Seven figures [file mmc1.pdf]

Comprehensive analysis of the green to blue photoconversion of full-length cyanobacteriochrome Tlr0924

Samantha J. O. Hardman,† Anna F. E. Hauck,† Ian P. Clark,‡ Derren J. Heyes,† Nigel S. Scrutton†*

†Manchester Institute of Biotechnology and Photon Science Institute, Faculty of Life Sciences, University of Manchester, 131 Princess Street, Manchester M1 7DN, UK

‡ Central Laser Facility, Research Complex at Harwell, Science and Technology Facilities Council, Harwell Oxford, Didcot, OX11 0QX, UK

S. J. O. Hardman and A. F. E. Hauck contributed equally to this work.

* Correspondence: nigel.scrutton@manchester.ac.uk

Supporting Material

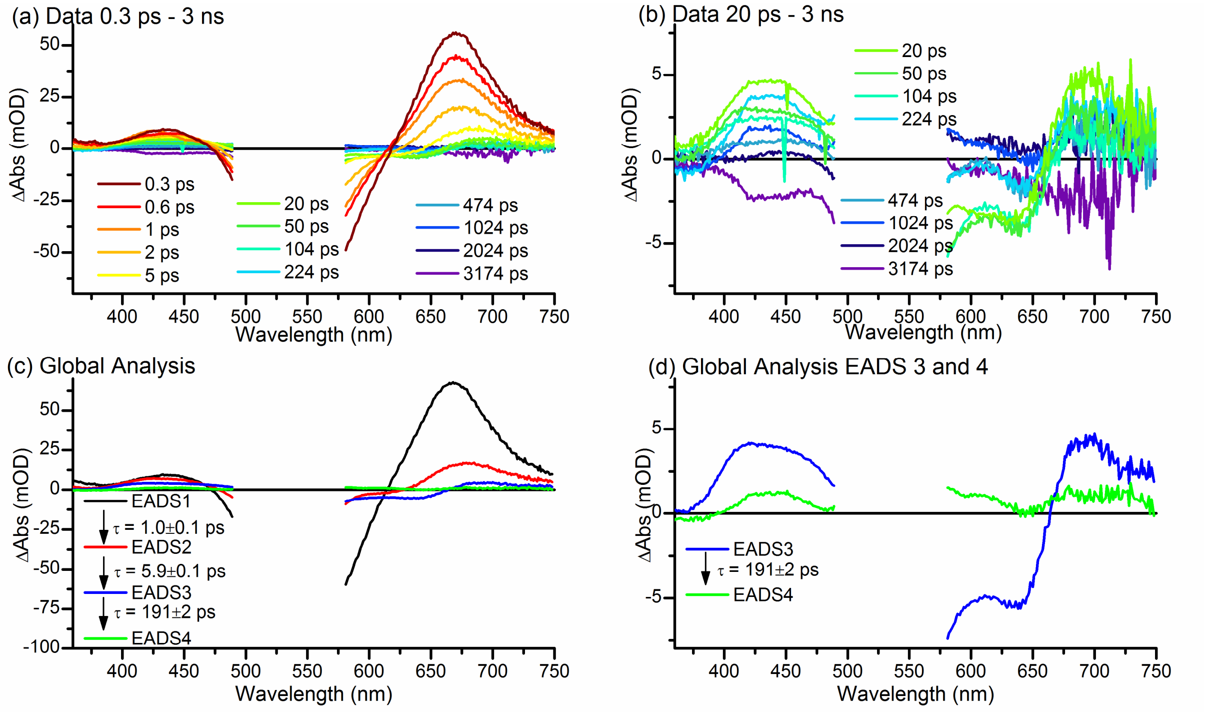

FIGURE S1 Ultrafast transient absorption spectra, collected using depolarized excitation with a wavelength of approximately 530nm, at selected time points for PVB Tlr0924 (a and b), global analysis of the ultrafast transient absorption data for PVB Tlr0924 (c and d)

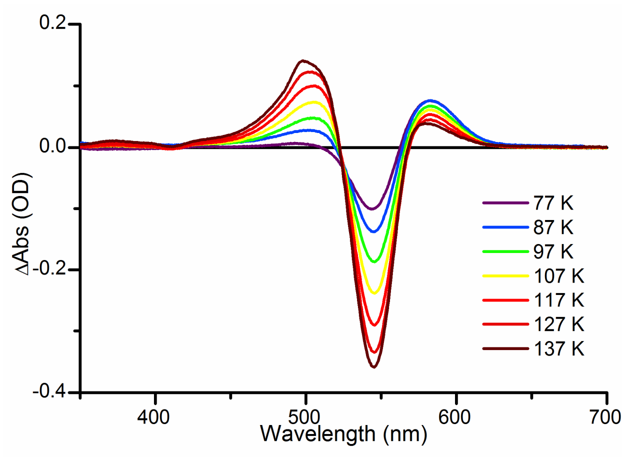

FIGURE S2 Cryotrapping experiments where the sample was warmed from 77 K in 10 K steps, illuminating at each temperature for 10 minutes before cooling to 77 K to record the spectrum

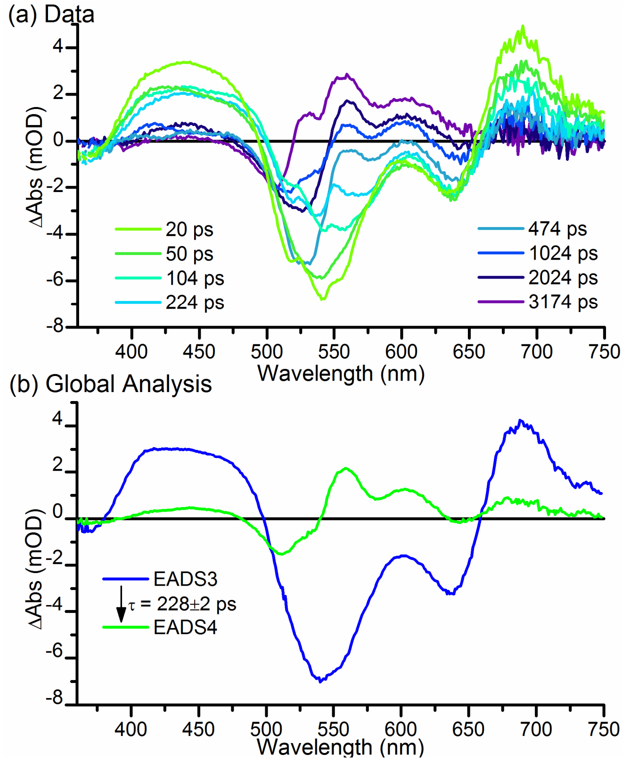

FIGURE S3 Ultrafast visible transient absorption spectra at time points between 20 and 3174 ps (a), and the second two EADS resulting from global analysis of the data (b). The sample was constantly illuminated during data collection with blue and red light so the signals primarily originate from the 'reverse' photoconversion of the PVB chromophore.

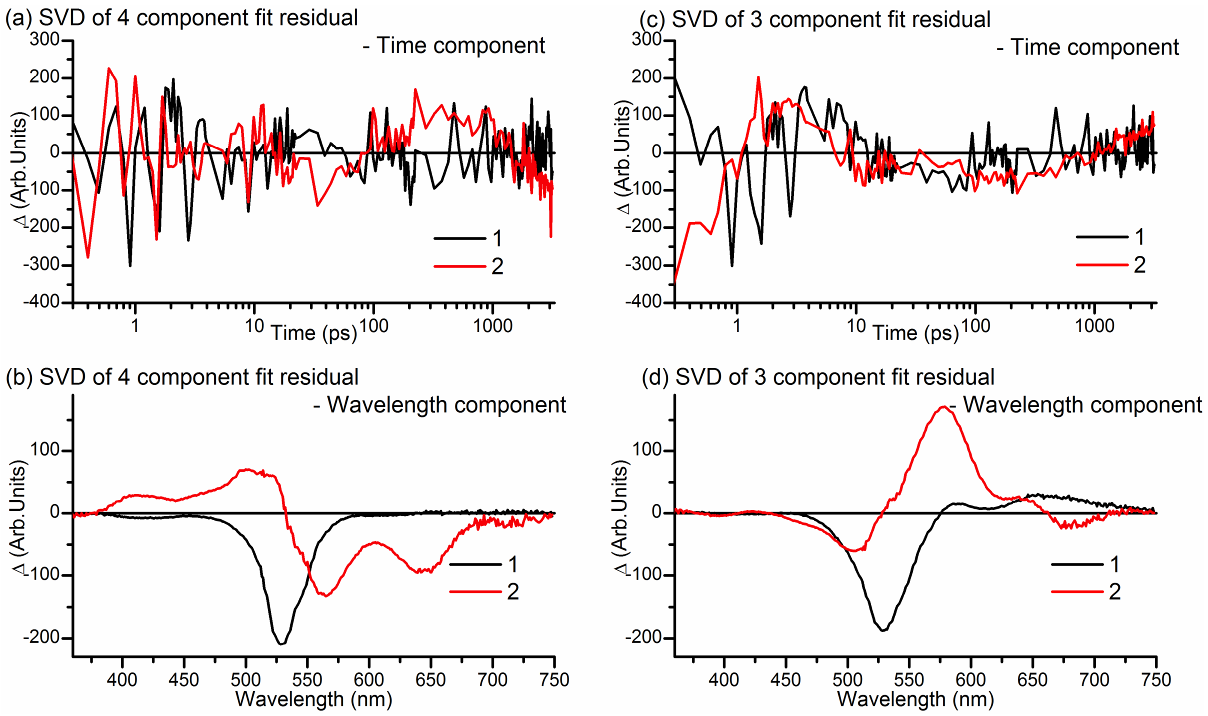

FIGURE S4 The residual matrix from the global analysis of the ultrafast transient absorption data is deconvolved by singular value decomposition to the component times (a) and wavelengths (b) . The corresponding analysis performed for a 3-component fit to the data is shown in figures (c) and (d) . The lack of obvious structure in the time-component of the 4-component fit residual implies a good fit has been obtained.

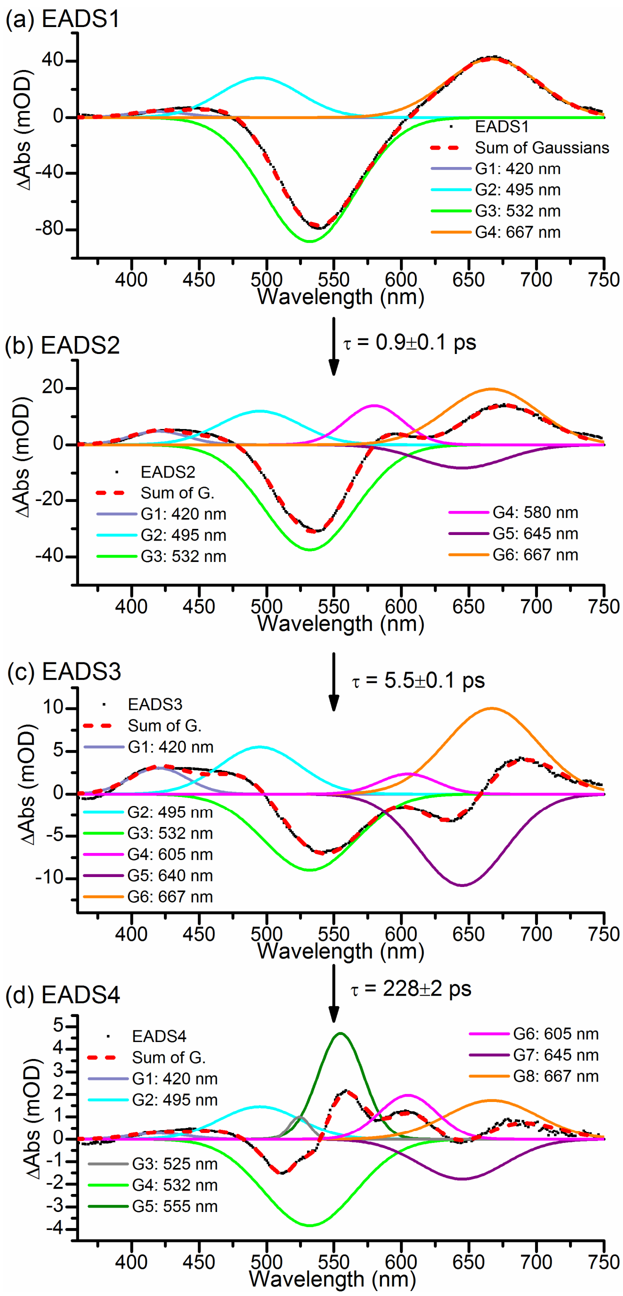

FIGURE S5 Sequential global analysis of the ultrafast visible transient absorption data showing resulting EADS (black dots) fitted with a sum of Gaussian functions (dashed red lines). The features are assigned as: GSB of the PVB Pg state at 532 nm (green line), ESA features at 420, 495, and 667 nm (lilac, cyan and orange lines), inactive or modified protein at 645 nm (purple line), intermediate states at 580, 605, and 555nm (pink and dark green), and pump scatter at 525 nm (grey line)

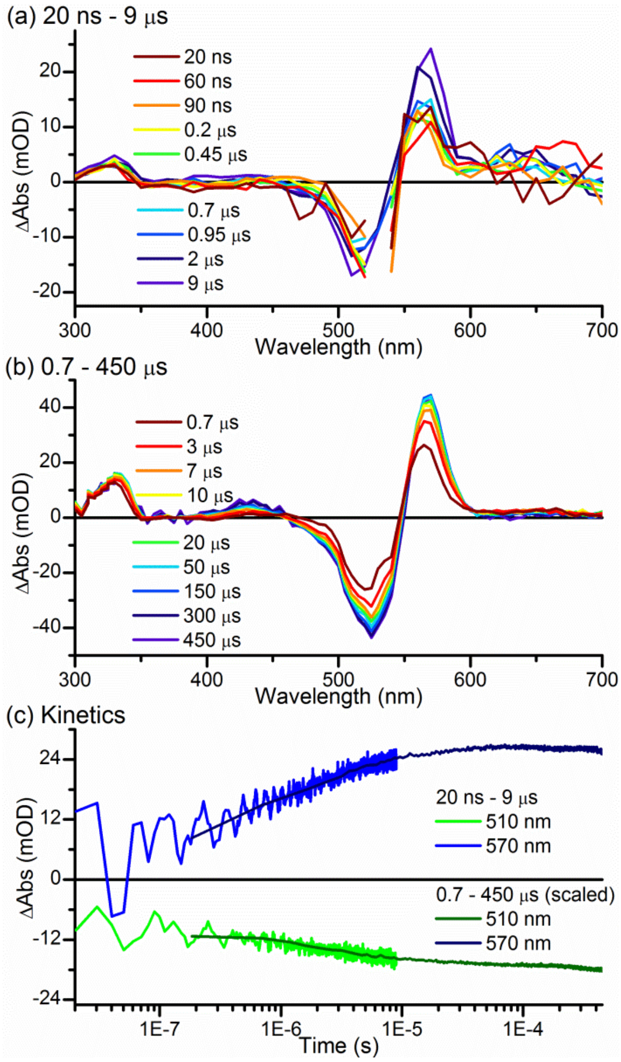

FIGURE S6 Laser flash photolysis data collected with PVB Tlr0924 after excitation at 532 nm: spectra at selected time points between 20 ns and 9 μs (a), and 0.7 and 450 μs (b), and kinetics at 510 and 570 nm (c).

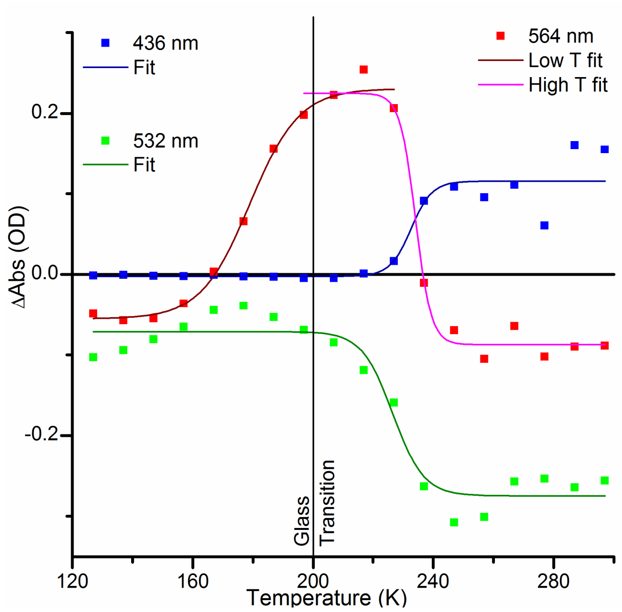

FIGURE S7 Temperature dependence of selected wavelengths over the 127 – 297 K temperature range fitted with Boltzmann distribution: $y = \frac{A_1 + A_2}{1 + e^{(x - x_0)/dx}} + A_2$ where A1 is the initial ΔAbs value, A2 is the final ΔAbs value, x_0 is the centre temperature, and dx is the 'time constant'. Values of x_0 are: 179±2 K and 234±1 K for the 564 nm data, 228±2 K for the 532 nm data, and 233±3 K for the 435 nm data.
