# Supplementary material for: Comprehensive Analysis of the Green-to-Blue Photoconversion of Full-Length Cyanobacteriochrome Tlr0924
Source: Biophys J. 2014 Nov 4;107(9):2195–203. doi: 10.1016/j.bpj.2014.09.020 (PMC4223177; doi:10.1016/j.bpj.2014.09.020)
Supplement: Document S2. Article plus Supporting Material [file mmc2.pdf]

Article

Comprehensive Analysis of the Green-to-Blue Photoconversion of Full-Length Cyanobacteriochrome Tlr0924

Samantha J. O. Hardman,[1] Anna F. E. Hauck,[1] Ian P. Clark,[2] Derren J. Heyes,[1] and Nigel S. Scrutton[1,*]
[1]Manchester Institute of Biotechnology and Photon Science Institute, Faculty of Life Sciences, University of Manchester, Manchester, UK; and [2]Central Laser Facility, Research Complex at Harwell, Science and Technology Facilities Council, Harwell Oxford, Didcot, UK

ABSTRACT Cyanobacteriochromes are members of the phytochrome superfamily of photoreceptors and are of central importance in biological light-activated signaling mechanisms. These photoreceptors are known to reversibly convert between two states in a photoinitiated process that involves a basic *E/Z* isomerization of the bilin chromophore and, in certain cases, the breakage of a thioether linkage to a conserved cysteine residue in the bulk protein structure. The exact details and timescales of the reactions involved in these photoconversions have not been conclusively shown. The cyanobacteriochrome Tlr0924 contains phycocyanobilin and phycoviolobilin chromophores, both of which photoconvert between two species: blue-absorbing and green-absorbing, and blue-absorbing and red-absorbing, respectively. Here, we followed the complete green-to-blue photoconversion process of the phycoviolobilin chromophore in the full-length form of Tlr0924 over timescales ranging from femtoseconds to seconds. Using a combination of time-resolved visible and mid-infrared transient absorption spectroscopy and cryotrapping techniques, we showed that after photoisomerization, which occurs with a lifetime of 3.6 ps, the phycoviolobilin twists or distorts slightly with a lifetime of 5.3 μs. The final step, the formation of the thioether linkage with the protein, occurs with a lifetime of 23.6 ms.

INTRODUCTION

Cyanobacteriochromes (CBCRs), a photochemically versatile class of proteins, mediate a variety of molecular outputs via the reversible *E/Z* isomerization of a sensory bilin chromophore in their cyanobacterial host organism (1). The general protein structure is divided into domains, which always include the photosensory chromophore binding GAF (cGMP-specific phosphodiesterase/adenylyl cyclase/FhlA protein) domain in the N-terminus and an output domain (e.g., histidine kinase or GGDEF domain) in the C-terminus (1,2). The different subgroups of this protein class show great functional versatility. Modifications of the central chromophore also permit a collective spanning of the entire UV/visible (UV/Vis) spectrum (1,3,4). CBCRs have attracted a great deal of attention as novel photoreceptors. Variations in the domain architecture and chromophore structure permit the creation of proteins with a specific function for applications such as fluorescence biomarking (5), optogenetics (6), and the production of biofuels (7). At the heart of these developments is a thorough understanding of the protein as a photoreceptor.

Submitted July 10, 2014, and accepted for publication September 24, 2014.

*Correspondence: nigel.scrutton@manchester.ac.uk

This is an open access article under the CC BY license (http://creativecommons.org/licenses/by/3.0/).

Samantha J. O. Hardman and Anna F. E. Hauck contributed equally to this work.

Editor: Leonid Brown.

© 2014 The Authors

0006-3495/14/11/2195/9 $2.00

CrossMark

The photochemical reactions of biological molecules are frequently fast and efficient. CBCRs and the related phytochromes and bacteriophytochromes are increasingly being characterized on femtosecond to nanosecond timescales (8–13). In general terms, both the forward *Z/E* isomerization and reverse *E/Z* isomerization of the chromophore usually occur within several hundred picoseconds (10,13,14). This induces further structural changes, manifested in the form of mostly unknown intermediate structures (15–17) and ultimately leading to protein conformational changes to activate the output domain. The CBCR Tlr0924 from *Thermosynechococcus elongatus* is a photoreceptor from the DXCF subgroup (containing the Asp-Xaa-Cys-Phe motif) of CBCRs, which has been the focus of a number of studies (18–21) and was recently found to regulate sessility in vivo (22). Heterologously expressed Tlr0924 incorporates a red-absorbing (Pr) phycocyanobilin (PCB) chromophore through covalent linkage between Cys-527 and the ethylidene side chain of ring A (Fig. 1) (21). On a timescale of days, the autoisomerase activity of the GAF domain subsequently converts ~80% of the PCB population to a phycoviolobilin (PVB) population (23,24). PVB is saturated at the C5 position, between rings A and B, and hence the π-conjugated system is restricted to rings B–D to yield a green-absorbing species (Pg). The *15Z* isomers of PVB and PCB can form a second covalent linkage between Cys-499 and the C10 position, between rings B and C, to form species PVB′ and PCB′, respectively. This second Cys linkage shortens the π-conjugation to rings C and D only in both

http://dx.doi.org/10.1016/j.bpj.2014.09.020

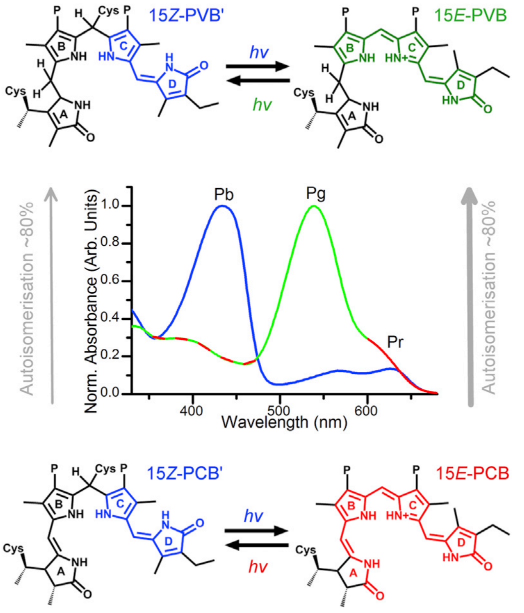

FIGURE 1 Structures and absorption spectra of the PCB and PVB photostates. The PCB and PVB chromophores can interconvert by autoisomerization, and the ^{15Z}Pb, ^{15E}Pg, and ^{15E}Pr states can reversibly photoconvert. To see this figure in color, go online.

chromophores, yielding identical blue-absorbing dark states (Pb). After photoconversion to the Pg or Pr states, reversion to the dark Pb states typically occurs slowly, over several hours (3,23). The combination of chromophores means that Tlr0924 is photosensitive to virtually the entire visible spectrum (Fig. 1). Previous studies have characterized the various photostates involved by using acid denaturation and selective sequential photoconversion (21,23). If Tlr0924 containing a mixture of PVB′ and PCB′ is illuminated with blue light, conversion will occur from both (spectrally identical in the visible region) Pb states. When the sample is then illuminated with red light only, the PCB Pr state will convert back to the Pb state. When green light is used, both the PVB and PCB populations will be returned to their respective Pb states.

A description of the complete forward photoconversion for the full-length protein was recently published (20). The only previous time-resolved study of the reverse photoreaction of Tlr0924 focused on the femtosecond-to-nanosecond photoisomerization dynamics of the GAF domain protein (18). Here, we use a combination of time-resolved spectroscopies (covering femtosecond-to-second timescales) and cryotrapping measurements to comprehensively characterize the reverse photoreaction of the dominant PVB chromophore in full-length Tlr0924.

MATERIALS AND METHODS

Protein expression and purification

Full-length Tlr0924 was expressed and purified as described previously (20). The sample was dissolved in a phosphate-based buffer system (100 mM sodium-potassium phosphate, 300 mM NaCl, pH 7). UV/Vis spectroscopy was used to determine the relative PVB/PCB chromophore ratio as ~80% PVB and 20% PCB. This ratio was assumed to remain constant over the course of the experiments (25).

Ultrafast transient absorption spectroscopy

The 1 kHz repetition rate laser system used to pump the broadband pump-probe visible transient absorbance spectrometer Helios (Ultrafast Systems LLC; Sarasota, FL) has been previously described (20). The pump beam was centered at 530 nm with a full width at half-maximum intensity of ~50 nm. Excitation energies of 0.6 μJ were used, yielding pump fluences of 3.4 mJ/cm^2. To maximize the accessible region of the spectrum, the polarization of the pump and probe was adjusted to be perpendicular, and a polarizer before the detectors was used to eliminate a large proportion of the scattered pump light. Data collected with a depolarized pump beam (see Fig. S1 in the Supporting Material) yielded kinetics and spectra similar to those shown in Fig. 2; thus, we assume that any polarization effects would not affect the model derived from these data. The time resolution of the experiment was ~0.2 ps and data points were collected randomly over the 3 ns time frame. Samples were contained in stirred 2-mm-pathlength quartz cuvettes (optical density (OD) at 535 nm = 0.5). During the measurements, the samples were continuously illuminated through the appropriate bandpass filter (Andover, Salem, NH) using a cold light source (KL1500; Schott, Stafford, UK). Illumination at 435 nm was used to regenerate the PVB and PCB Pr and Pg states from their corresponding Pb states, and simultaneous 640 nm illumination was used to regenerate the PCB Pb state from the Pr state. This left only the PVB Pg state to be excited by the pump laser.

Time-resolved infrared (IR) spectroscopy was carried out at the Ultra Facility (Central Laser Facility, STFC Rutherford Appleton Laboratory, Didcot, UK), which uses a 10 kHz repetition rate laser and has a time resolution of ~100 fs (26). Samples dissolved in D_2O-based phosphate buffer (at the same concentration and equivalent pD as the H_2O buffer used in the visible measurements) were flowed through a 100-μm-pathlength CaF_2 measurement cell (Harrick Scientific, Pleasantville, NY) and the sample holder was rastered in the two dimensions orthogonal to the pump and probe beams to avoid sample damage. The sample was concentrated by a factor of ~20 times compared with that used in the visible measurements, so the OD at 535 nm was ~0.5. An excitation energy of 1 μJ at 530 nm was used and the pump beam diameter was ~150 μm, yielding a fluence of 7.2 mJ/cm^2. The excitation beam was set at the magic angle with respect to the IR probe beam. The Pg state of the sample was regenerated by continuous sample illumination with a cold light source as described above. The spectral resolution was ~3 cm^{-1} and pixel-to-wavenumber calibration was performed as described previously (27).

Laser flash photolysis

The laser flash photolysis experimental setup has been described in detail elsewhere (20). For measurements on submillisecond timescales, the probe beam was pulsed and kinetic traces were recorded on a digital oscilloscope (Infiniium, 54830B; Agilent Technologies, Santa Clara, CA). Measurements on longer timescales were recorded using a photomultiplier tube. The 532 nm pump pulse duration was 6–8 ns and energies of 26–100 mJ (depending on the set of measurements) were used. The beam diameter was on the order of 1 cm, yielding pump fluences of 33–128 mJ/cm^2. The 300–700 nm region was monitored by recording absorption transients in 5 nm steps, with each data point being the average of three or more transients. The PVB Pg photostate was regenerated after each shot by illumination with a cold source lamp fitted with the relevant bandpass filters. Illumination at 435 nm was used to regenerate the PVB and PCB Pr and Pg states from their corresponding Pb states, followed by a 640 nm illumination that regenerated the PCB′ Pb state from the Pr state. This left only the PVB Pg state to be excited by the pump laser. The sample was contained in

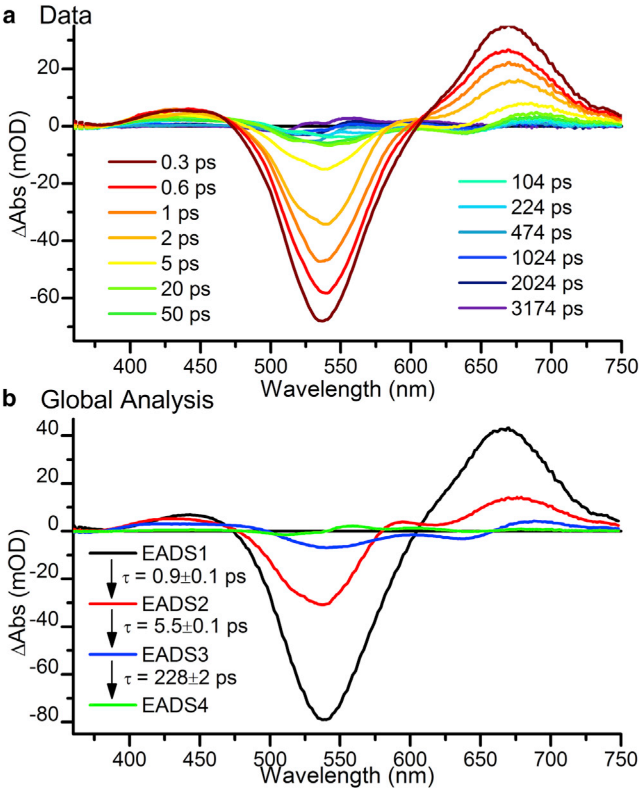

FIGURE 2 (*a*) Ultrafast visible TA spectra at selected time points. (*b*) EADS resulting from a sequential global analysis of the data. The sample was constantly illuminated during data collection with blue and red light, so the signals primarily originate from the Pg-to-Pb photoconversion of the PVB chromophore. To see this figure in color, go online.

a 1 cm pathlength quartz cuvette and maintained at 25°C by a circulating water bath. The sample had an OD of 0.5 at the excitation wavelength. Samples were frequently replaced and their quality was monitored by UV/Vis spectroscopy (Cary 50; Agilent Technologies).

Cryotrapping

Samples were prepared in the PVB Pg photostate as described above, with an OD at 435 nm of ~1 in cryogenic buffer (100 mM sodium-potassium phosphate, 300 mM NaCl, pH 7, and an aqueous buffer system made with 30% glycerol and 48% sucrose) and cooled to 99 K in a cryochamber (Optistat DN liquid nitrogen cryostat; Oxford Instruments, Abingdon, UK). After a UV/Vis reference spectrum (Cary 50; Agilent Technologies) was recorded at 99 K, the sample was illuminated at 127 K for 10 min before being cooled back to 99 K. The sample was then warmed up to 297 K in 10 K steps. At each point, the sample was equilibrated for ~10 min before it was cooled to 99 K, and a UV/Vis absorbance spectrum was recorded. A temperature of 127 K was chosen as the illumination temperature because this was the point at which the ground-state bleach (GSB) feature reached the maximum intensity in a set of benchmarking experiments in which the sample was warmed from 77 K in 10 K steps and illuminated at each temperature for 10 min before being cooled to 77 K to record the spectrum (see Fig. S2).

Global analysis

Transient absorption (TA) and laser flash photolysis 3D data sets were analyzed globally using the open-source software Glotaran (28). This procedure reduces the matrix of time, wavelength, and change in absorbance to one or more exponentially decaying time components, each with a corresponding difference spectrum. The visible and IR ultrafast TA data were fitted from 0.3 and 0.2 ps, respectively, to avoid any contributions from coherent artifacts (29).

RESULTS AND DISCUSSION

Ultrafast TA

To isolate the initial photoreaction of the PVB chromophore in Tlr0924, the samples were illuminated simultaneously with constant blue and red light and excited with a ~530 nm pulsed laser as described above. The autoisomerization of chromophores in Tlr0924, which occurs on a timescale of days, results in an approximate PVB/PCB ratio of 4:1 (24). The comparatively small population of the PCB chromophore combined with the selective wavelength constant illumination allows almost complete isolation of the PVB photodynamics. The ultrafast TA data collected for the initial steps of the PVB photoconversion are shown in Fig. 2 *a*. Within the time resolution of the experiment (0.2 ps), two major spectral features appear: a large negative feature at ~530 nm, which can be assigned to the GSB of the Pg state, and a positive feature at ~670 nm, which can be assigned to the excited-state absorption (ESA) of the Pg state. Within 5 ps, these features have decayed almost completely, although they are all still distinguishable and a positive feature at ~550 nm appears (see Fig. S3). We carried out a global analysis to more accurately define the spectral intermediates and the timescales of interconversion between intermediate states (Fig. 2 *b*). A model with four sequentially converting spectral components, the evolution-associated difference spectra (EADS), was found to be a good fit to the data (see Fig. S4) and broadly agreed with literature values for GAF-domain-only Tlr0924 (18). There was also an obvious contribution from a negative feature at ~645 nm (see Fig. S5), which was previously ascribed to inactive or modified protein (20,21).

We collected ultrafast IR TA data over the same time range, using the same time steps employed for the visible TA experiments. The data shown in Fig. 3 *a* display a relatively simple picture. There are three major negative features at 1402, 1606, and 1685 cm^{-1}, but no immediately apparent positive features. Similar phytochromes have been extensively studied in the mid-IR region, allowing assignment of the 1606 and 1685 cm^{-1} bleaches as C=C stretches in rings A and B (30–32), and C=O stretches in ring D (33,34), respectively. Global analysis of the data set using a basic sequential model yields time constants comparable to those found from the visible TA data (Fig. 3 *b*). One less component is required to fit the data, and the resulting time constants are 3.6, 196, and infinite picoseconds.

To more accurately model the ultrafast processes that occur upon photoisomerization, we performed a more

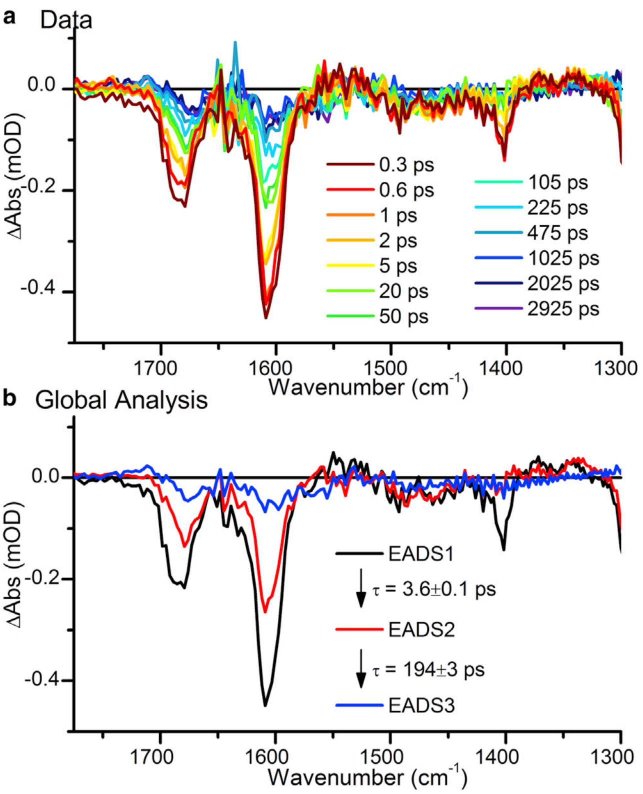

FIGURE 3 (*a*) Ultrafast IR TA spectra at selected time points. (*b*) EADS resulting from a sequential global analysis of the data. The sample was constantly illuminated during data collection with blue and red light, so the signals primarily originate from the Pg-to-Pb photoconversion of the PVB chromophore To see this figure in color, go online.

complex global analysis (Fig. 4) to produce species-associated difference spectra (SADS). This model was selected after many iterations because it was the model that not only isolated the negative feature at ~645 nm but also resulted in very comparable lifetimes between the visible and IR data sets. In this model, SADS1 evolves into a nondecaying component, SADS2. Independently and in parallel with this process, SADS3, representing the inactive protein, decays back to the ground state. In the case of the visible TA data, an additional lifetime representing SADS1 decaying to the ground state was also fitted. The lifetimes produced by this model correlate extremely well with the visible and IR TA data sets. The SADS1-to-SADS2 conversion lifetime was 3.6 $\pm$ 0.1 ps in both data sets. The lifetime of SADS3 was fitted as 185 $\pm$ 2 ps and 194 $\pm$ 2 ps for the visible and IR TA data, respectively. In the analysis of the visible TA, SADS1 was found to relax to the ground state with a lifetime of 0.6 $\pm$ 0.1 ps. This component was not resolved in the IR TA data, likely due to the similarity between the excited-state and ground-state vibrational spectra, and with the poor signal/noise ratio of these data playing a role. The poor signal/noise ratio was due to both the low volume sample and air bubbles in the sample, which were unavoidable due to the high flow rate that was necessary to avoid sample damage during the measurements.

A combination of Gaussian peaks were fitted to the SADS resulting from the visible TA data (Fig. 4, *a–c*). Although it has been shown that bilin chromophores do not have pure Gaussian lineshapes, the use of these functions can provide useful insight into which species are present in each spectrum (35,36). SADS1 is very similar in shape to the difference spectra collected at 0.3 ps after excitation. Features corresponding to the PVB Pg GSB centered at 532 nm, as well as ESA features at 440, 496, and 663 nm, are clearly defined. The lack of any other negative features in this spectrum is consistent with excitation of only the Pg state, and not of any intermediate states that may be present after 1 ms (the separation of pulses in the 1 kHz laser system). These features remain in SADS2, which displays an additional positive feature, which we ascribe to the first reaction intermediate at 555 nm. There are also less intense components at 525, 604, and 648 nm, which may be real features but are more likely to be artifacts from scattered pump light, residual signal from the inactive protein, and the very low signal levels. SADS3 decays in parallel with the SADS1-to-SADS2 conversion and has features similar (but not identical) to those of SADS1 and SADS2 at 407, 438, 483, 554, 581, and 685 nm. The most significant difference is the large negative feature at 641 nm, which corresponds to inactive or modified protein as observed previously (20). This feature does not correspond to any known state of PCB or PVB in Tlr0924 (23), and various other PCB-containing CBCRs show features in this spectral region (16,37), suggesting that it is a variation in the GAF domain that produces this red-shifted, inactive population of the protein. This population may account for some of the spectral density above 600 nm in the Pb absorption spectrum shown in Fig. 1. The SADS resulting from analysis of the IR TA data support the species assignments of the visible TA analysis. SADS1 displays the three major bleach peaks observed in the raw data. In addition to the features observed in SADS1, there is a small but significant positive feature at ~1711 cm^{-1} in SADS2. This correlates well with ultrafast IR TA measurements on the forward photoconversion of PVB′ from the Pb state, where shortly after photoexcitation, a downshift is observed in the same C=O stretching feature (from ~1700 to ~1686 cm^{-1}), which is assigned to the isomerization reaction (20). In parallel with the SADS1 to SADS2 conversion, SADS3 decays with a lifetime of 194 ps. This spectrum confirms the assignment of this independently decaying component as a structurally modified version of the protein. Although SADS1 and SADS2 do appear to be similar, SADS3 completely lacks the bleach at ~1402 cm^{-1}, and the C=O stretching feature at ~1686 cm^{-1} in SADS1 appears to have downshifted to ~1680 cm^{-1}.

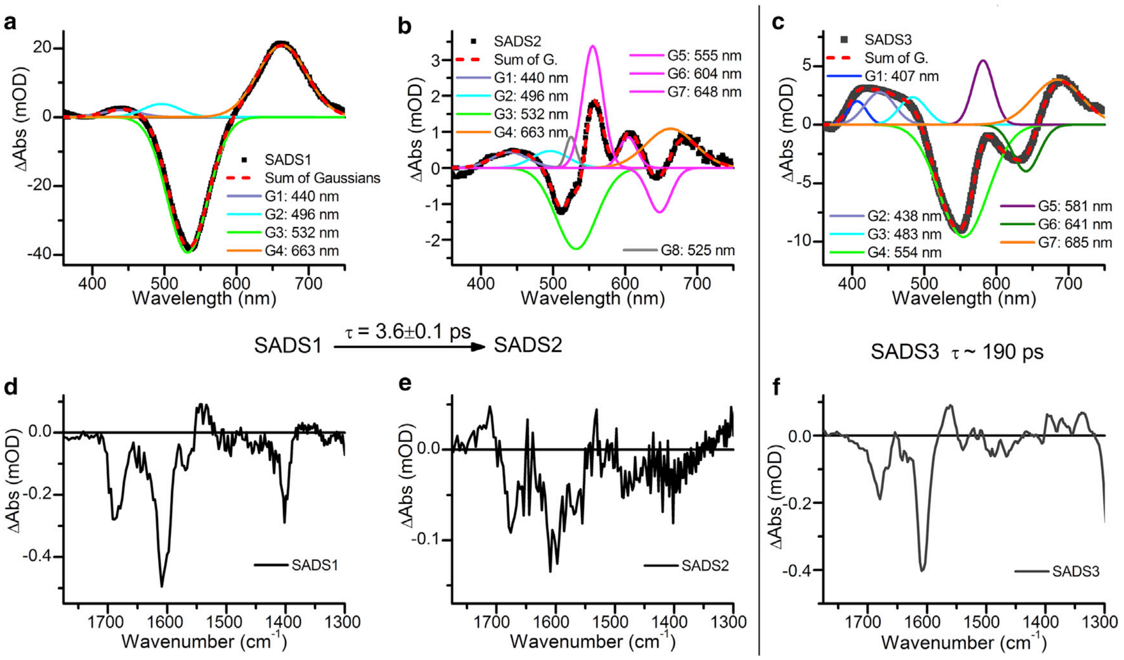

FIGURE 4 (*a–f*) Global analysis of the ultrafast visible (*a–c*) and IR (*d–f*) TA data, showing the resulting SADS (*black*). The visible TA SADS are fitted with a sum of Gaussian functions (*dashed red*). The features of SADS1 (*a*) and SADS2 (*b*) are assigned as follows: GSB of the PVB Pg state at 532 nm (*green*); ESA features at 440, 496, and 663 nm (*lilac*, *cyan*, and *orange*); intermediate states at 550, 604, and 648 nm (*pink*); and pump scatter at 525 nm (*gray*). SADS3, representing inactive or modified protein, has additional peaks at 641 nm (*dark green*) and 581 nm (*purple*). To see this figure in color, go online.

Nanosecond-to-millisecond TA

We used laser flash photolysis to monitor the slower processes that occurred in the photoinitiated reaction. By illuminating the entire sample with blue light, to produce the Pg and Pr states, and then illuminating with red light, we were able to completely isolate the Pg species. To cover the nanosecond-to-millisecond time range, we performed two sets of measurements: from 20 ns to 8 μs, and from ~1 μs to 450 μs. From the data shown in Figs. 5 and S6, it is evident that both spectral and kinetic changes occur in the PVB over these timescales.

Because the submicrosecond data set did not show any additional features, we analyzed the 1–450 μs data set using a sequential model (Fig. 5, *b–d*). Three components were needed to completely describe the data. The first component, EADS1, is qualitatively similar to the SADS2 derived from the ultrafast visible TA measurements (Fig. 4 *b*). The laser flash photolysis measurements extend further into the UV, allowing further characterization of the various intermediates. The significant features present in both difference spectra are the GSBs at 532 (and 340 nm), and the intermediate with a strong absorbance at 555 (and 332) nm. The less-intense features appear to be slightly different (440, 496, 604, and 663 nm in the ultrafast data, compared with 442 and 650 nm in the flash-photolysis data). Any differences can be attributed to differences in the experimental techniques used. Flash photolysis data will show all changes compared with a true pre-excitation signal. In contrast, the ultrafast visible and IR pump-probe measurements use a 1 or 10 kHz laser, respectively, so the data will show changes compared with the sample 1 or 0.1 ms after excitation. EADS1 decays into EADS2 with a lifetime of 5.3 μs, and EADS3 grows in from EADS2 with a lifetime of 387 μs. The major spectral features of EADS2 and EADS3 are identical: the GSB features at 340 and 532 nm remain, but the intermediate features that were previously at 332 and 555 nm shift to 335 and 564 nm, respectively. The difference between EADS2 and EADS3 lies in the lower-intensity features in the red region of the spectrum, which are present in EADS2, but not EADS3. This may be due to a small proportion of the PCB Pr-to-Pb conversion occurring in parallel with the much more intense PVB processes. The final intermediate in these data, with an absorption maximum at 564 nm, corresponds exactly to the intermediate that was previously observed in non-time-resolved studies and was assigned by those studies to the isomerized but not yet thioether-linked chromophore (21,23).

Millisecond-to-second TA

The final set of laser flash photolysis measurements on millisecond timescales monitored the final step in the photoconversion reaction, when the isomerized intermediate forms a thioether linkage with the Cys-499 residue in

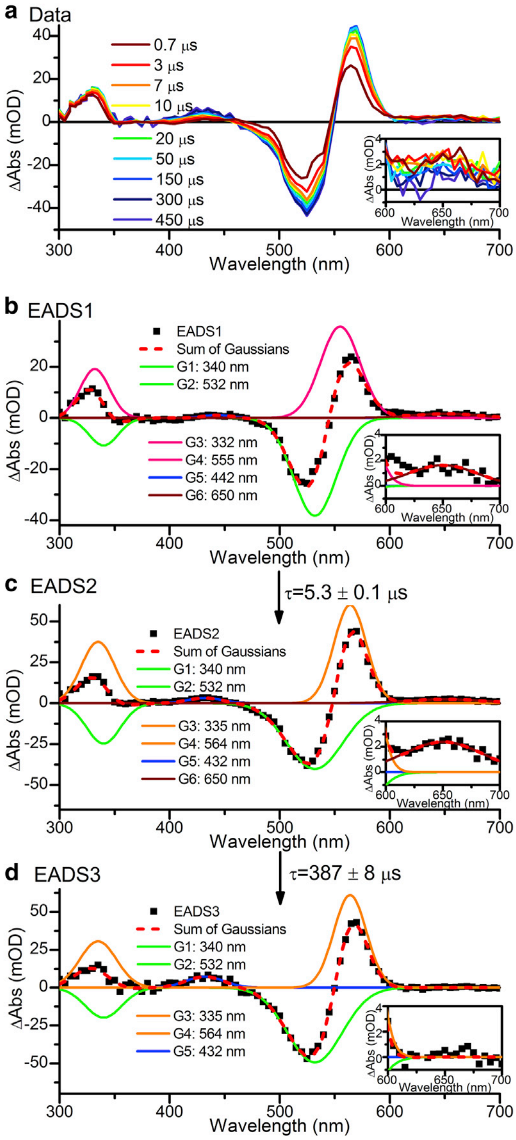

FIGURE 5 (*a*) TA data for PVB Tlr0924 after excitation at 532 nm collected over 1–450 μs. (*b–d*) EADS resulting from global analysis (*black dots*) fitted with a sum of Gaussian functions (*dashed red*). The features are assigned as follows: GSB of the PVB Pg state at 532 and 340 nm (*green*), the first intermediate at 555 and 332 nm (*pink*), and the second intermediate at 564 and 335 nm (*orange*). Insets show an expanded 600–700 nm region containing the 650 nm intermediate (*maroon*). To see this figure in color, go online.

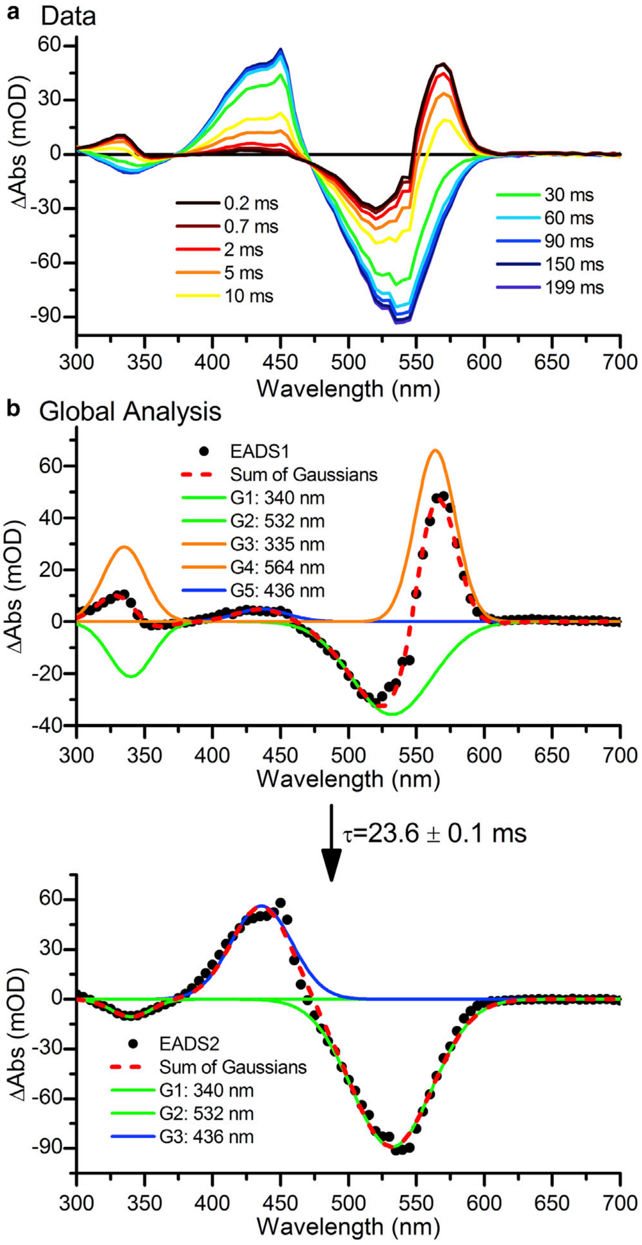

FIGURE 6 (*a*) TA spectra of PVB Tlr0924 after excitation at 532 nm at selected time points over 0.2–199 ms. (*b*) Global analysis of the data, showing the resulting EADS (*black dots*) fitted with a sum of Gaussian functions (*red line*). There are features originating from the bleach of the Pg state (*green lines*), the second intermediate (*orange lines*), and the final Pb state (*blue lines*). To see this figure in color, go online.

the protein. The PVB Pg population was isolated as described in the previous section. The data, shown in Fig. 6 *a*, initially have the same features as those described in the final EADS of the nanosecond-to-millisecond data set. Over the course of a few milliseconds, this converts to the final state with a large positive feature at ~430 nm. The data fit well to a sequential model with only two components, which interconvert with a lifetime of 23.6 ms. The first EADS is essentially identical to EADS3 in Fig. 5 *d*, with the GSB of the Pg state at 340 and 532 nm, and the intermediate with features at 335 and 564 nm. EADS1 converts to the final difference spectra, EADS2, in which the GSB of the Pg state remains, and the final Pb state at 436 nm is apparent.

Cryotrapping

To determine which of the transitions observed in the time-resolved measurements were thermally activated, we carried

out cryotrapping measurements. In these experiments, a sample in the PVB Pg state was illuminated with a cold light source fitted with a 530 nm bandpass filter at 127 K and then warmed to 297 K in 10 K steps, with spectra collected at 99 K between each temperature point. Difference spectra relative to the dark spectrum at 99 K are shown in Fig. 7, *a* and *b*.

The difference spectra in Fig. 7 *a* show a number of intermediates being formed. Throughout the measurements, the GSB at ~530 nm is visible. At the lowest temperatures, there are positive features visible at ~583 nm and ~506 nm that are lost at 157 and 177 K, respectively. These two features are not observed in the time-resolved measurements, where the GSB is significantly more intense due to the large excited-state population. Whereas the ~506 nm feature reduces in intensity as the sharp feature at ~560 nm appears, the ~583 nm peak does not seem to correlate with any other features. There are two most likely explanations for these features. They may be artifacts of the cryogenic techniques used, since previous studies have shown that cryogenic temperatures can affect the energy landscape and possible conformational substates of proteins (38–40). This variation in protein structure could in turn affect the absorption properties of the chromophore. The other possibility is that these features are true intermediates in the photoreaction, which were not resolvable in the ultrafast measurements because of either their very short lifetimes (<<0.6 ps) or low intensity relative to the very strong excited-state features.

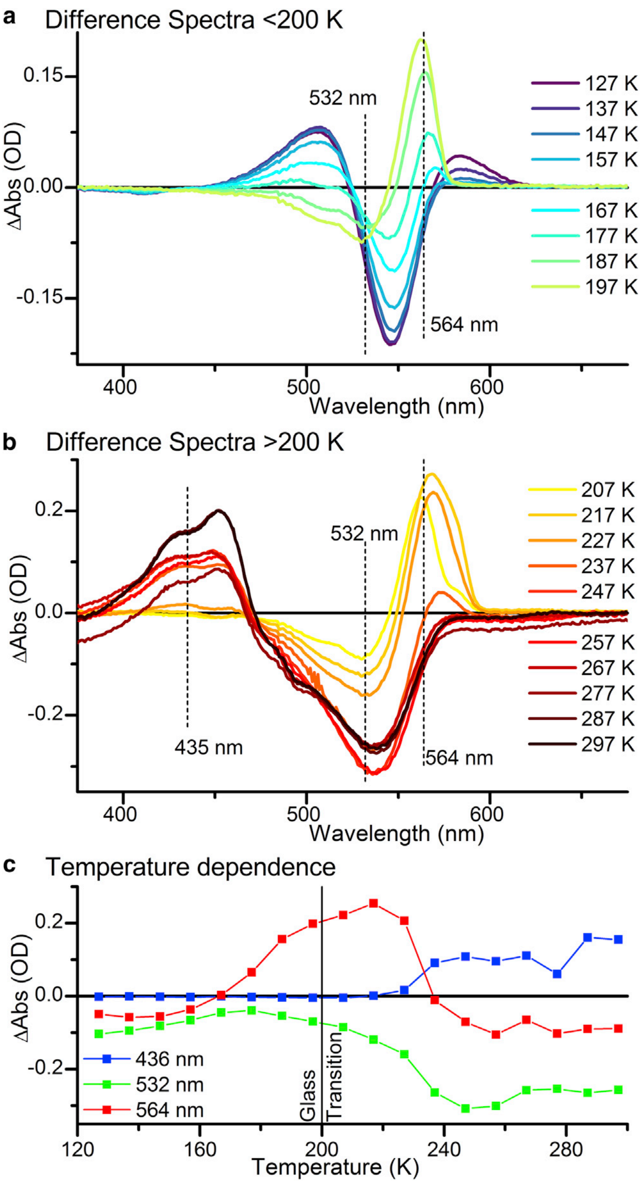

FIGURE 7 (*a* and *b*) Difference spectra at selected temperatures relative to the dark spectrum at 99 K (*a*) below 200 K and (*b*) above 200 K. (*c*) Temperature dependence of selected wavelengths over the 127–297 K temperature range. To see this figure in color, go online.

Above ~187 K (Fig. 7 *b*), the spectral features in the cryotrapping data more closely resemble the features observed in the time-resolved data. The temperature-dependent spectral evolution at selected wavelength points is displayed in Fig. 7 *c*. It is not possible to resolve the 555 and 564 nm intermediates, but the final step in the time-resolved data from the intermediate at 564 nm to the Pb state correlates very well with the final step observed in the cryotrapping measurements at 237 K. By fitting the temperature dependence at selected wavelengths (Fig. S7), one can put the temperature of formation of the 555/564 nm intermediate at ~179 K and the formation of the final product state at ~232 K. It is known that at ~200 K, proteins undergo a dynamic transition, termed the glass transition, below which any large-scale conformational changes in the protein that require solvent reorganization become frozen out (9,41). As the formation of the 555/564 nm intermediate occurs close to this temperature, it may be accompanied by some minor structural changes in the protein. However, the final transition to form the Pb state can only proceed well above this glass transition temperature, demonstrating that more large-scale protein motions are likely to be required for this stage of the photoconversion. This picture is similar to that observed in the forward reaction of Tlr0924 and the photoreaction of the related Cph1 phytochrome (9,20).

CONCLUSIONS

The time-resolved visible and IR TA data described here present a clear picture of the PVB Pg-to-Pb photoconversion (Fig. 8). After photoexcitation of the PVB Pg state, the majority of the excited-state population relaxes back to the ground state with a lifetime of 0.6 ps. A small proportion of the excited population isomerizes with a lifetime of 3.6 ps to the 15*Z*-PVB, with an absorption maximum at 555 nm. The final Pb state of the PVB′ is linked by a thioether linkage to a Cys in the bulk protein structure. Before this linkage is formed, another step, with a lifetime of 5.3 μs, occurs to form a further intermediate absorbing at 564 nm. The

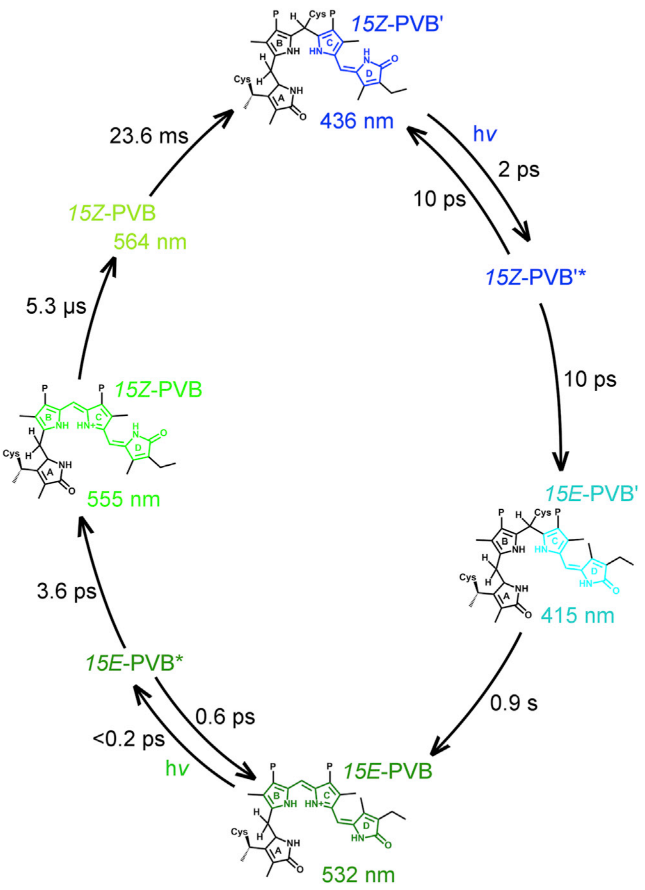

FIGURE 8 Suggested reaction scheme for the full PVB conversion cycle, including details of both the Pg-to-Pb reaction from this work and the Pb-to-Pg reaction (20). To see this figure in color, go online.

structure of this intermediate is not certain, but it involves a red shift in the absorbance maxima at temperatures close to the glass transition temperature of proteins. Hence, it is possible that it may involve some twisting or other structural distortion of the chromophore, assisted by protein motions in the binding pocket, in which it becomes more planar, delocalizing the π-orbital system and causing the red shift in the absorbance maxima (16). Similar intermediates have been suggested for a number of related phytochrome and CBCR systems (16,42–45). The final step of the reaction progresses with a lifetime of 23.6 ms to the final Cys-bound 15*Z*-Pb state and likely involves some large-scale movement of the protein structure.

The time-resolved data presented here for microsecond-to-millisecond timescales are comparable to those reported for other CBCRs. Flash photolysis measurements have shown that the slower steps in the photoconversion process often proceed via two or three intermediates with spectrally distinct features. The precise nature of these intermediates is not confirmed, but isomerization and changes in the localization of the π-orbital system have been suggested. There is quite a variation in the lifetimes of conversion between these intermediates. The final two steps can occur with lifetimes of ~1 μs and ~920 μs (AnPixJ) (16), 750 ns and >1 ms (NpF2164g3) (19), and 390 μs and 1.5 ms (Slr1393) (46). None of these lifetimes are as long as the 23.6 ms observed for the final reaction step reported here, even in the case of NpF2164g3, which, like Tlr0924, forms two thioether linkages with the protein. However, the above-cited studies focused on the GAF-domain-only system, whereas in this work we investigated the full-length protein, which may significantly extend the kinetics (46).

The scheme suggested here is more complex than that suggested for the Pb-to-Pg forward reaction (20), which involves only two steps: fast photoisomerization followed by very slow breakage of the thioether linkage. Neither of those steps depends strongly on the geometry and configuration of the chromophore ring systems. In contrast, in the reverse reaction after photoisomerization, the chromophore must move into a geometry that is favorable for formation of the thioether linkage, but overall the reaction progresses more rapidly and is completed in milliseconds rather than seconds.

SUPPORTING MATERIAL

Seven figures are available at http://www.biophysj.org/biophysj/supplemental/S0006-3495(14)00994-1.

This work was funded by the Engineering and Physical Sciences Research Council (EPSRC; reference numbers EP/I01974X/1 and EP/J020192/1). N.S.S. is an EPSRC Established Career Fellow and a Royal Society Wolfson Merit Awardee. The time-resolved IR measurements were carried out through program access support from the UK Science and Technology Facilities Council (STFC). A.F.E.H. was funded by a Biotechnology and Biological Sciences Research Council Collaborative Award in Science and Engineering (BBSRC CASE) award supported by TgK Scientific Ltd. (Bradford-On-Avon, UK).

REFERENCES

1. Ikeuchi, M., and T. Ishizuka. 2008. Cyanobacteriochromes: a new superfamily of tetrapyrrole-binding photoreceptors in cyanobacteria. *Photochem. Photobiol. Sci.* 7:1159–1167.
2. Narikawa, R., T. Ishizuka, …, M. Ikeuchi. 2013. Structures of cyanobacteriochromes from phototaxis regulators AnPixJ and TePixJ reveal general and specific photoconversion mechanism. *Proc. Natl. Acad. Sci. USA.* 110:918–923.
3. Hirose, Y., N. C. Rockwell, …, M. Ikeuchi. 2013. Green/red cyanobacteriochromes regulate complementary chromatic acclimation via a protochromic photocycle. *Proc. Natl. Acad. Sci. USA.* 110:4974–4979.
4. Rockwell, N. C., S. S. Martin, …, J. C. Lagarias. 2011. Diverse two-cysteine photocycles in phytochromes and cyanobacteriochromes. *Proc. Natl. Acad. Sci. USA.* 108:11854–11859.
5. Sun, Y.-F., J.-G. Xu, …, M. Zhou. 2014. Orange fluorescent proteins constructed from cyanobacteriochromes chromophorylated with phycoerythrobilin. *Photochem. Photobiol. Sci.* 13:757–763.
6. Pathak, G. P., J. D. Vrana, and C. L. Tucker. 2013. Optogenetic control of cell function using engineered photoreceptors. *Biol. Cell.* 105:59–72.
7. Zhou, J., and Y. Li. 2010. Engineering cyanobacteria for fuels and chemicals production. *Protein Cell.* 1:207–210.

8. Linke, M., Y. Yang, …, K. Heyne. 2013. Electronic transitions and heterogeneity of the bacteriophytochrome Pr absorption band: an angle balanced polarization resolved femtosecond VIS pump-IR probe study. *Biophys. J.* 105:1756–1766.
9. Heyes, D. J., B. Khara, …, N. S. Scrutton. 2012. Ultrafast red light activation of Synechocystis phytochrome Cph1 triggers major structural change to form the Pfr signalling-competent state. *PLoS ONE.* 7:e52418.
10. Müller, M. G., I. Lindner, …, A. R. Holzwarth. 2008. Femtosecond kinetics of photoconversion of the higher plant photoreceptor phytochrome carrying native and modified chromophores. *Biophys. J.* 94: 4370–4382.
11. Heyne, K., J. Herbst, …, R. Diller. 2002. Ultrafast dynamics of phytochrome from the cyanobacterium synechocystis, reconstituted with phycocyanobilin and phycoerythrobilin. *Biophys. J.* 82:1004–1016.
12. Gottlieb, S. M., P. W. Kim, …, D. S. Larsen. 2013. Primary photodynamics of the green/red-absorbing photoswitching regulator of the chromatic adaptation E domain from Fremyella diplosiphon. *Biochemistry.* 52:8198–8208.
13. Bischoff, M., G. Hermann, …, D. Strehlow. 2001. First steps in the phytochrome phototransformation: a comparative femtosecond study on the forward (Pr —> Pfr) and back reaction (Pfr —> Pr). *Biochemistry.* 40:181–186.
14. Kim, P. W., L. H. Freer, …, D. S. Larsen. 2012. Femtosecond photodynamics of the red/green cyanobacteriochrome NpR6012g4 from Nostoc punctiforme. 2. Reverse dynamics. *Biochemistry.* 51:619–630.
15. Burgie, E. S., J. M. Walker, …, R. D. Vierstra. 2013. A photo-labile thioether linkage to phycoviolobilin provides the foundation for the blue/green photocycles in DXCF-cyanobacteriochromes. *Structure.* 21:88–97.
16. Fukushima, Y., M. Iwaki, …, S. Itoh. 2011. Photoconversion mechanism of a green/red photosensory cyanobacteriochrome AnPixJ: time-resolved optical spectroscopy and FTIR analysis of the AnPixJ-GAF2 domain. *Biochemistry.* 50:6328–6339.
17. Piwowarski, P., E. Ritter, …, F. Bartl. 2010. Light-induced activation of bacterial phytochrome Agp1 monitored by static and time-resolved FTIR spectroscopy. *ChemPhysChem.* 11:1207–1214.
18. Freer, L. H., P. W. Kim, …, D. S. Larsen. 2012. Chemical inhomogeneity in the ultrafast dynamics of the DXCF cyanobacteriochrome Tlr0924. *J. Phys. Chem. B.* 116:10571–10581.
19. Gottlieb, S. M., P. W. Kim, …, D. S. Larsen. 2014. Primary and secondary photodynamics of the violet/orange dual-cysteine NpF2164g3 cyanobacteriochrome domain from Nostoc punctiforme. *Biochemistry.* 53:1029–1040.
20. Hauck, A. F. E., S. J. O. Hardman, …, N. S. Scrutton. 2014. The photoinitiated reaction pathway of full-length cyanobacteriochrome Tlr0924 monitored over 12 orders of magnitude. *J. Biol. Chem.* 289:17747–17757.
21. Rockwell, N. C., S. L. Njuguna, …, S. C. Spiller. 2008. A second conserved GAF domain cysteine is required for the blue/green photoreversibility of cyanobacteriochrome Tlr0924 from Thermosynechococcus elongatus. *Biochemistry.* 47:7304–7316.
22. Enomoto, G., R. Nomura, …, M. Ikeuchi. 2014. Cyanobacteriochrome SesA is a diguanylate cyclase that induces cell aggregation in Thermosynechococcus. *J. Biol. Chem.* 289:24801–24809.
23. Rockwell, N. C., S. S. Martin, …, J. C. Lagarias. 2012. Phycoviolobilin formation and spectral tuning in the DXCF cyanobacteriochrome subfamily. *Biochemistry.* 51:1449–1463.
24. Ishizuka, T., A. Kamiya, …, M. Ikeuchi. 2011. The cyanobacteriochrome, TePixJ, isomerizes its own chromophore by converting phycocyanobilin to phycoviolobilin. *Biochemistry.* 50:953–961.
25. Rockwell, N. C., Y. S. Su, and J. C. Lagarias. 2006. Phytochrome structure and signaling mechanisms. *Annu. Rev. Plant Biol.* 57:837–858.
26. Greetham, G. M., P. Burgos, …, M. Towrie. 2010. ULTRA: a unique instrument for time-resolved spectroscopy. *Appl. Spectrosc.* 64:1311–1319.
27. Jones, A. R., H. J. Russell, …, N. S. Scrutton. 2012. Ultrafast infrared spectral fingerprints of vitamin B12 and related cobalamins. *J. Phys. Chem. A.* 116:5586–5594.
28. Snellenburg, J. J., S. P. Laptenok, …, I. H. M. van Stokkum. 2012. Glotaran: a Java-based graphical user interface for the R Package TIMP. *J. Stat. Softw.* 49:1–22.
29. Lorenc, M., M. Ziolek, …, A. Maciejewski. 2002. Artifacts in femtosecond transient absorption spectroscopy. *Appl. Phys. B.* 74:19–27.
30. Salewski, J., F. V. Escobar, …, P. Hildebrandt. 2013. Structure of the biliverdin cofactor in the Pfr state of bathy and prototypical phytochromes. *J. Biol. Chem.* 288:16800–16814.
31. Mroginski, M. A., F. Mark, …, P. Hildebrandt. 2007. Quantum mechanics/molecular mechanics calculation of the Raman spectra of the phycocyanobilin chromophore in α-C-phycocyanin. *Biophys. J.* 93:1885–1894.
32. Dasgupta, J., R. R. Frontiera, …, R. A. Mathies. 2009. Ultrafast excited-state isomerization in phytochrome revealed by femtosecond stimulated Raman spectroscopy. *Proc. Natl. Acad. Sci. USA.* 106: 1784–1789.
33. Toh, K. C., E. A. Stojković, …, J. T. M. Kennis. 2011. Primary reactions of bacteriophytochrome observed with ultrafast mid-infrared spectroscopy. *J. Phys. Chem. A.* 115:3778–3786.
34. van Thor, J. J., K. L. Ronayne, and M. Towrie. 2007. Formation of the early photoproduct lumi-R of cyanobacterial phytochrome cph1 observed by ultrafast mid-infrared spectroscopy. *J. Am. Chem. Soc.* 129:126–132.
35. Mirkovic, T., A. B. Doust, …, G. D. Scholes. 2007. Ultrafast light harvesting dynamics in the cryptophyte phycocyanin 645. *Photochem. Photobiol. Sci.* 6:964–975.
36. Zehetmayer, P., M. Kupka, …, A. Zumbusch. 2004. Energy transfer in monomeric phycoerythrocyanin. *Biochim. Biophys. Acta.* 1608:35–44.
37. Rockwell, N. C., S. S. Martin, and J. C. Lagarias. 2012. Red/green cyanobacteriochromes: sensors of color and power. *Biochemistry.* 51:9667–9677.
38. Anderson, S., V. Šrajer, and K. Moffat. 2004. Structural heterogeneity of cryotrapped intermediates in the bacterial blue light photoreceptor, photoactive yellow protein. *Photochem. Photobiol.* 80:7–14.
39. Frauenfelder, H. 2010. Proteins, supercooled liquids, and glasses: a micro-review. *Physica E.* 42:662–665.
40. Lubchenko, V., P. G. Wolynes, and H. Frauenfelder. 2005. Mosaic energy landscapes of liquids and the control of protein conformational dynamics by glass-forming solvents. *J. Phys. Chem. B.* 109:7488–7499.
41. Vitkup, D., D. Ringe, …, M. Karplus. 2000. Solvent mobility and the protein 'glass' transition. *Nat. Struct. Biol.* 7:34–38.
42. Yoshihara, S., T. Shimada, …, S. Tokutomi. 2006. Reconstitution of blue-green reversible photoconversion of a cyanobacterial photoreceptor, PixJ1, in phycocyanobilin-producing Escherichia coli. *Biochemistry.* 45:3775–3784.
43. Matysik, J., P. Hildebrandt, …, K. Schaffner. 1995. Fourier-transform resonance Raman spectroscopy of intermediates of the phytochrome photocycle. *Biochemistry.* 34:10497–10507.
44. Foerstendorf, H., C. Benda, …, F. Siebert. 2001. FTIR studies of phytochrome photoreactions reveal the C=O bands of the chromophore: consequences for its protonation states, conformation, and protein interaction. *Biochemistry.* 40:14952–14959.
45. Rockwell, N. C., S. S. Martin, …, J. C. Lagarias. 2014. Conserved phenylalanine residues are required for blue-shifting of cyanobacteriochrome photoproducts. *Biochemistry.* 53:3118–3130.
46. Xu, X.-L., A. Gutt, …, W. Gärtner. 2014. Combined mutagenesis and kinetics characterization of the bilin-binding GAF domain of the protein Slr1393 from the Cyanobacterium Synechocystis PCC6803. *ChemBioChem.* 15:1190–1199.

Comprehensive analysis of the green to blue photoconversion of full-length cyanobacteriochrome Tlr0924

Samantha J. O. Hardman,[†] Anna F. E. Hauck,[†] Ian P. Clark,[‡] Derren J. Heyes,[†] Nigel S. Scrutton[†]*

[†]Manchester Institute of Biotechnology and Photon Science Institute, Faculty of Life Sciences, University of Manchester, 131 Princess Street, Manchester M1 7DN, UK

[‡] Central Laser Facility, Research Complex at Harwell, Science and Technology Facilities Council, Harwell Oxford, Didcot, OX11 0QX, UK

S. J. O. Hardman and A. F. E. Hauck contributed equally to this work.

* Correspondence: nigel.scrutton@manchester.ac.uk

Supporting Material

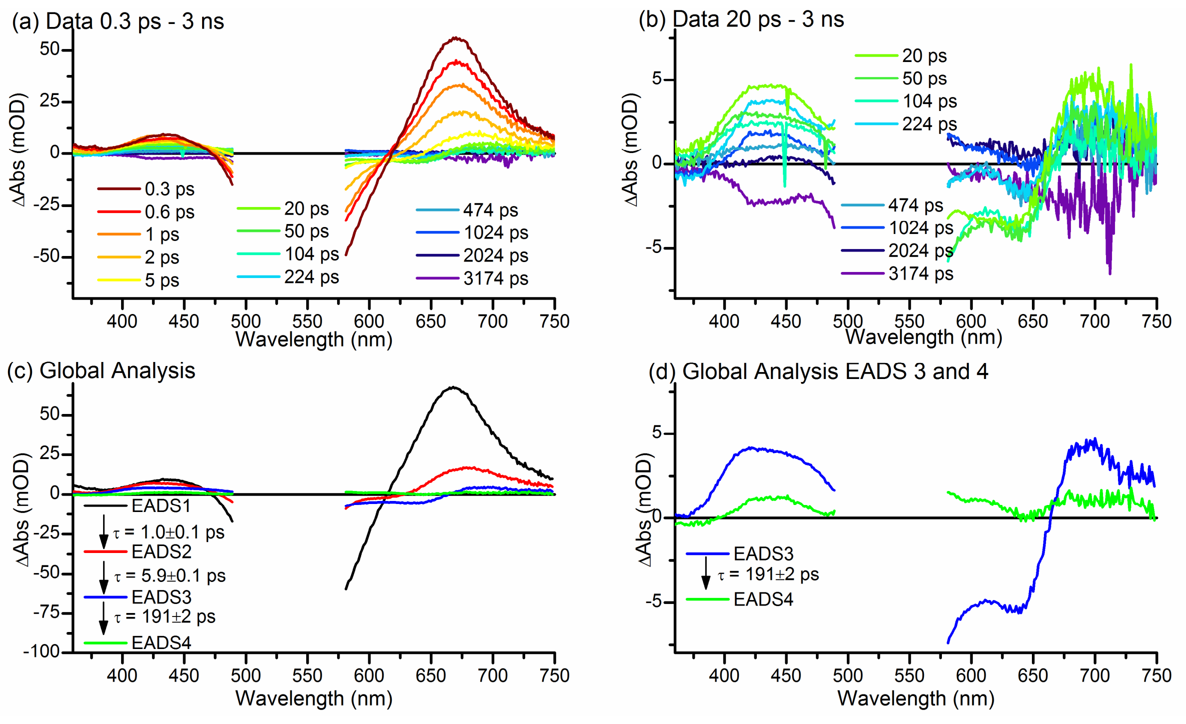

FIGURE S1 Ultrafast transient absorption spectra, collected using depolarized excitation with a wavelength of approximately 530nm, at selected time points for PVB Tlr0924 (a and b), global analysis of the ultrafast transient absorption data for PVB Tlr0924 (c and d)

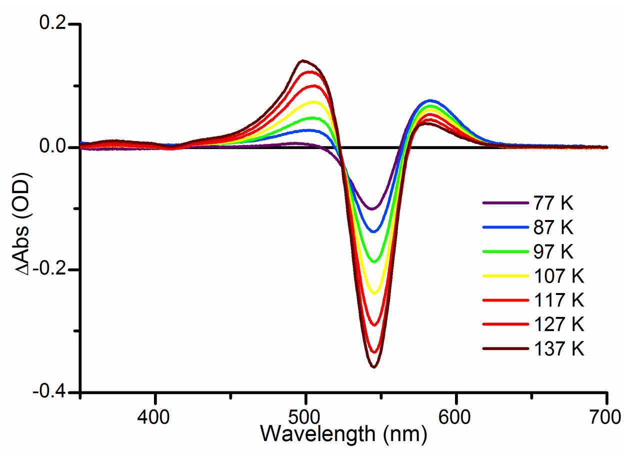

FIGURE S2 Cryotrapping experiments where the sample was warmed from 77 K in 10 K steps, illuminating at each temperature for 10 minutes before cooling to 77 K to record the spectrum

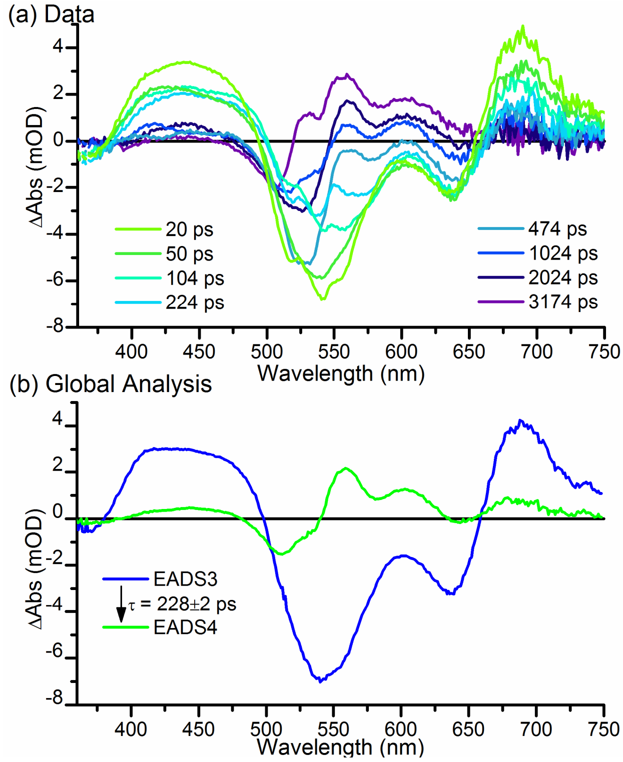

FIGURE S3 Ultrafast visible transient absorption spectra at time points between 20 and 3174 ps (a), and the second two EADS resulting from global analysis of the data (b). The sample was constantly illuminated during data collection with blue and red light so the signals primarily originate from the 'reverse' photoconversion of the PVB chromophore.

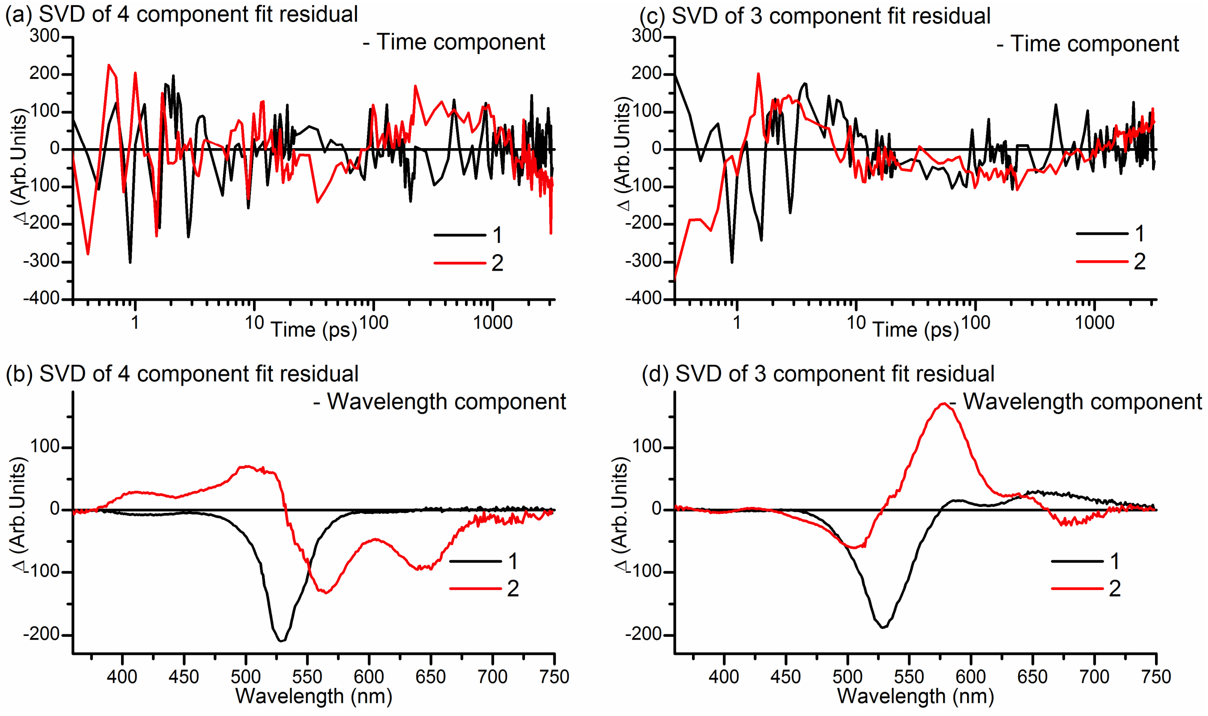

FIGURE S4 The residual matrix from the global analysis of the ultrafast transient absorption data is deconvolved by singular value decomposition to the component times (a) and wavelengths (b) . The corresponding analysis performed for a 3-component fit to the data is shown in figures (c) and (d) . The lack of obvious structure in the time-component of the 4-component fit residual implies a good fit has been obtained.

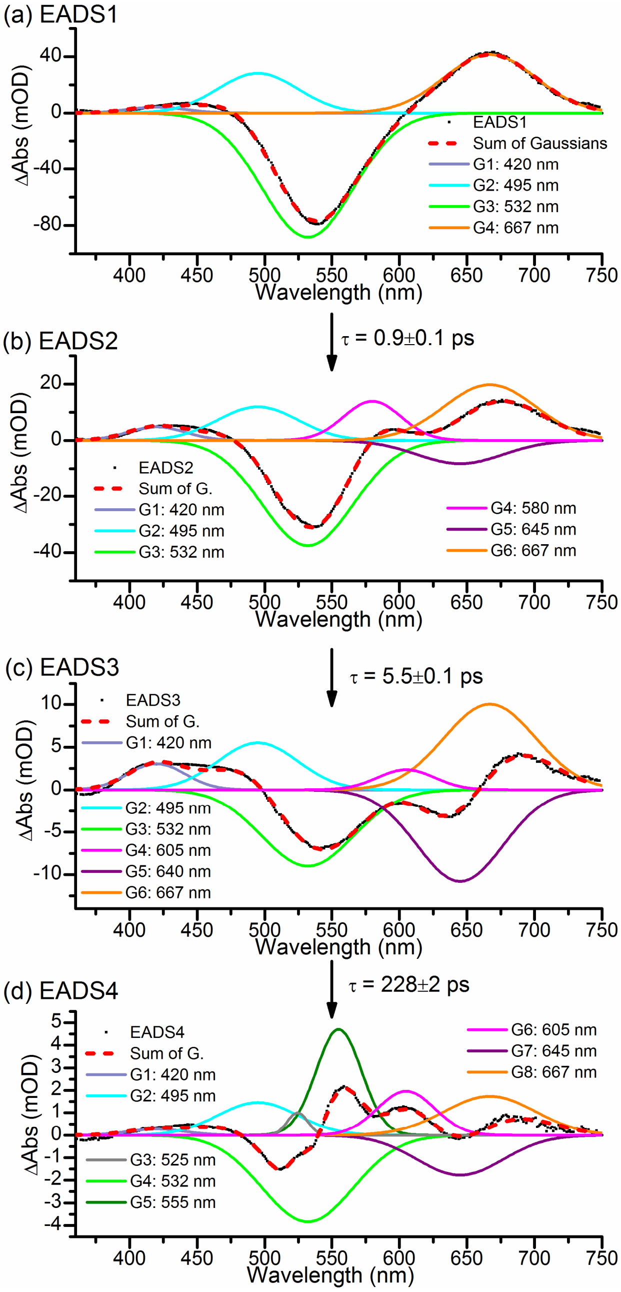

FIGURE S5 Sequential global analysis of the ultrafast visible transient absorption data showing resulting EADS (black dots) fitted with a sum of Gaussian functions (dashed red lines). The features are assigned as: GSB of the PVB Pg state at 532 nm (green line), ESA features at 420, 495, and 667 nm (lilac, cyan and orange lines), inactive or modified protein at 645 nm (purple line), intermediate states at 580, 605, and 555nm (pink and dark green), and pump scatter at 525 nm (grey line)

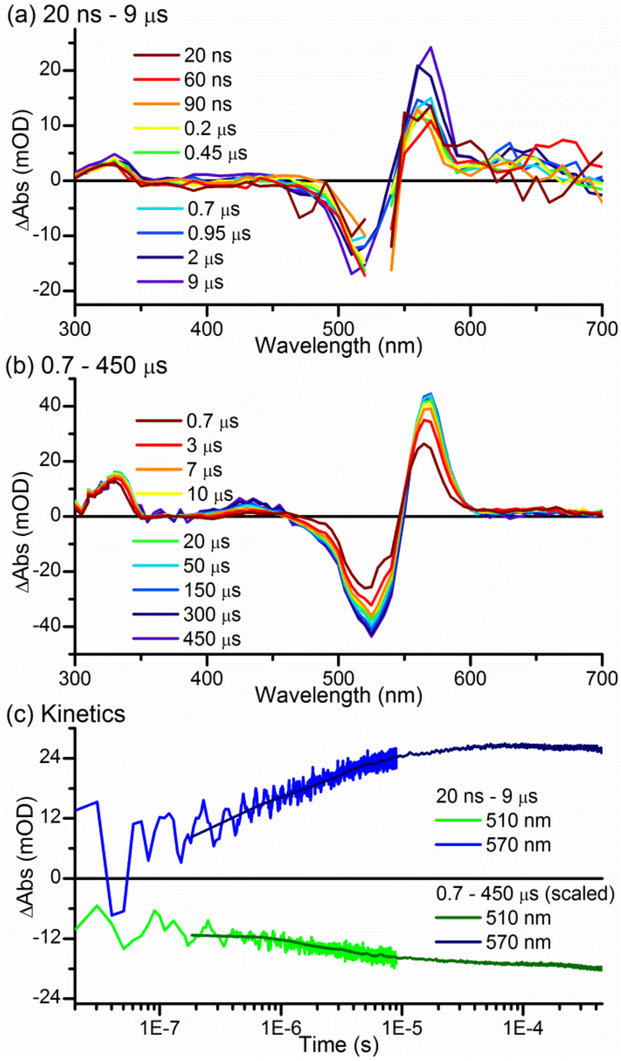

FIGURE S6 Laser flash photolysis data collected with PVB Tlr0924 after excitation at 532 nm: spectra at selected time points between 20 ns and 9 µs (a), and 0.7 and 450 µs (b), and kinetics at 510 and 570 nm (c).

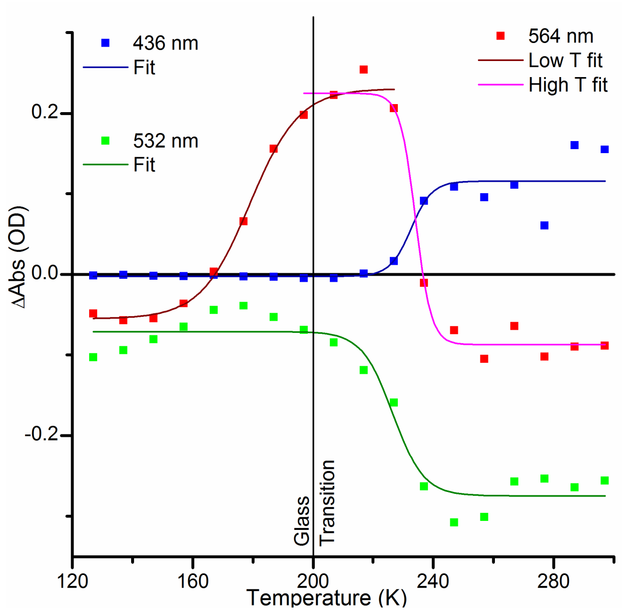

FIGURE S7 Temperature dependence of selected wavelengths over the 127 – 297 K temperature range fitted with Boltzmann distribution: $y = \frac{A_1 + A_2}{1 + e^{(x - x_0)/dx}} + A_2$ where A1 is the initial ΔAbs value, A2 is the final ΔAbs value, x_0 is the centre temperature, and dx is the 'time constant'. Values of x_0 are: 179±2 K and 234±1 K for the 564 nm data, 228±2 K for the 532 nm data, and 233±3 K for the 435 nm data.
